# Risk Factors for Residual Stenosis After Carotid Artery Stenting

**DOI:** 10.3389/fneur.2020.606924

**Published:** 2021-01-28

**Authors:** Yunlu Tao, Yang Hua, Lingyun Jia, Liqun Jiao, Beibei Liu

**Affiliations:** ^1^Department of Vascular Ultrasonography, Xuanwu Hospital, Capital Medical University, Beijing, China; ^2^Center of Vascular Ultrasonography, Beijing Institute of Brain Disorders, Beijing, China; ^3^Department of Neurosurgery, Xuanwu Hospital, Capital Medical University, Beijing, China

**Keywords:** carotid stenosis, stent, ultrasound, residual stenosis, risk factors

## Abstract

**Background and purpose:** Stent residual stenosis is an independent risk factor for restenosis after stenting. This study aimed to analyze the factors influencing residual stenosis after carotid artery stenting (CAS).

**Methods:** A total of 570 patients who underwent CAS with 159 closed-loop stents (CLS) and 411 open-loop stents (OLS) from January 2013 to January 2016 were retrospectively enrolled in this study. Carotid stenosis location in the common carotid artery or in internal carotid artery, plaque size, and features (regular or irregular morphology; with or without calcification), degree of carotid artery stenosis, and stent expansion rate were detected by carotid duplex ultrasonography. Residual stenosis was defined as a stenosis rate ≥30% after CAS, as detected by digital subtraction angiography. A logistic regression analysis was used to analyze residual stenosis risk factors.

**Results:** The overall incidence of residual stenosis was 22.8% (130/570 stents). The incidence of residual stenosis in the CLS group was higher than that in the OLS group (29.5 vs. 20.2%, χ^2^ = 5.71, *P* = 0.017). The logistic regression analysis showed that CLS [odds ratio (OR), 1.933; 95% confidence interval (CI), 1.009–3.702], irregular plaques (OR, 4.237; 95% CI, 2.391–7.742), and plaques with calcification (OR, 2.370; 95% CI, 1.337–4.199) were independent risk factors for residual stenosis after CAS. In addition, a high radial expansion rate of stent was a protective factor for residual stenosis (OR, 0.171; 95% CI, 0.123–0.238). The stenosis location and stent length did not impact the occurrence of residual stenosis. After 1-year follow-up, the incidence of restenosis in the residual stenosis group was higher than that in the group without residual stenosis (13.1 vs. 2.0%, χ^2^ = 28.05, *P* < 0.001).

**Conclusions:** The findings of this study suggest that plaque morphology, echo characteristics (with calcification), and stents type influence residual stenosis.

## Introduction

Carotid stenosis or occlusion is one of the main causes of ischemic stroke. Carotid artery stenting (CAS) has become an alternative treatment for symptomatic carotid stenosis and has emerged as a possible alternative to carotid endarterectomy (CEA) (1). Recent studies have shown that the rates of early restenosis after CAS are lower than those of CEA; nevertheless, due to the short follow-up period of many published studies, the long-term durability of CAS needs further investigation (2). Multiple trials have compared the two treatment modalities in the past. With the recently completed CEA vs. CAS trial (3, 4), there is a resurgence of interest in CAS.

Restenosis after CAS is an important factor that affects long-term efficacy. Prior studies have reported that the rate of restenosis after CAS varies from 5 to 11% over different follow-up periods (5). A recent systemic review of 27 studies found that the >50% restenosis rate after CAS was 18.21% at >1 year (21 months on average) of follow-up. Residual stenosis after stenting was an independent risk factor for restenosis (6–8). Few studies have focused on the risk factors for residual stenosis after CAS. In this study, plaque characteristics and carotid stenosis hemodynamic parameters before and after stenting were analyzed. The stent expansion rate (SER) was detected by carotid duplex ultrasonography (CDU). Finally, the factors that influence residual stenosis after CAS were determined using a logistic regression.

## Materials and Methods

### Subjects

**Figure 1:**
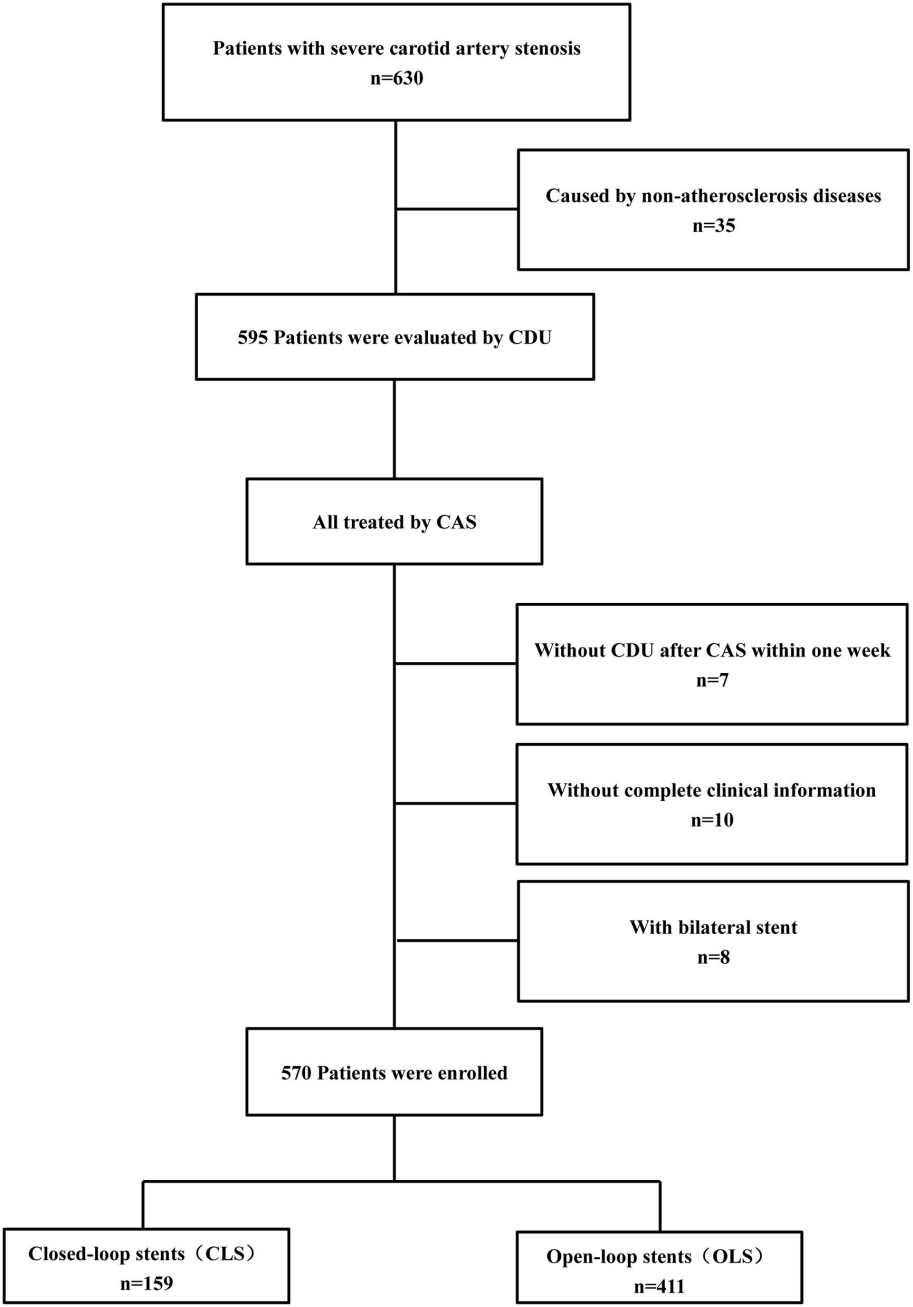
The flow diagram of patient enrollment. Study flow chart depicting all patients enrolled in the study as well as events precluding patients from this analysis.

This single-center retrospective study was approved by the Institutional Review Board of Xuanwu Hospital, Capital Medical University. Xuanwu Hospital is a senior stroke center in China that is renowned for cerebrovascular disease diagnosis and treatment. From January 2013 to January 2016, a total of 630 hospitalized patients were diagnosed with carotid stenosis or occlusion in the Department of Neurosurgery, Xuanwu Hospital. According to the following inclusion and exclusion criteria, 570 patients were enrolled for our analysis, and the study flow diagram is shown in Figure 1.

Inclusion criteria: patients (age range 41–89 years) with severe (70–99%) carotid stenosis caused by atherosclerosis diseases detected by CDU and confirmed by digital subtraction angiography (DSA).

Exclusion criteria: (1) patients with severe carotid artery stenosis caused by non-atherosclerosis diseases, such as dissection or arteritis (*n* = 35); (2) patients who received bilateral CAS (*n* = 8); (3) patients without complete clinical or imaging information (*n* = 10); (4) patients not evaluated by CDU before CAS or within 1 week after CAS (*n* = 7).

Finally, a total of 570 patients were enrolled in this study, 159 of whom were treated with closed-loop stents (CLS) and 411 with open-loop stents (OLS).

### Carotid Duplex Ultrasonography

CDU was performed within 1 week before and after CAS, and patients were followed-up for different periods (3, 6, 12, 18, 24, and 36 months) after CAS. The evaluation and detection of CDU was based on the vascular ultrasonography guidelines published by the Chinese Medical Doctor Association of Ultrasonography (9). Philips IU-Elite (Philips Medical Systems, Amsterdam, The Netherlands) and Hitachi Ascendus (Hitachi, Inc., Tokyo, Japan) ultrasound instruments were adopted, and an ultra-wideband probe of 3.0–9.0 MHz and a microconvex probe of 4.0–8.0 MHz were selected. All ultrasound examinations were performed by senior vascular ultrasound physicians with ≥5 years of experience.

The CDU pre-CAS plaque imaging and parameters were stored in PACS and analyzed later. The information included: (1) the position of stenosis distal to the CCA and/or proximal internal carotid artery (ICA). (2) Plaque characteristics, such as morphology (regular or irregular) and echogenic features. According to the distinction of calcified plaques, plaques can be divided into four categories: surface, bottom, interior, and non-calcification (10). (3) The residual and original carotid artery diameters at the stenosis segment (11).

The post-CAS imaging and parameters included: (1) the length (L) of the stent, divided into types L1 (< 4.0 cm), L2 (4.0 cm), and L3 (>4.0 cm). (2) The inner stent diameter, PSV, and EDV of the proximal, middle, and distal stent segments, depending on the stent length. The SER was calculated as the radial expansion rate (RER) (%) = (preoperative stenosis rate – residual stenosis rate poststenting) × 100%; the preoperative stenosis rate (%) = (1 – residual diameter / original diameter) × 100%. The residual stenosis rate was calculated in the same way as the preoperative diameter stenosis rate. Axial expansion rate (AER) of stent (%) = (the stent length detected by CDU / the initial stent length) × 100%. (3) Stent restenosis was defined as stenosis ≥50% at more than 3 months after CAS.

### CAS Implantation

New infarctions were not detected within 3 weeks by computed tomography (CT) or magnetic resonance imaging (MRI). The procedure was performed according to the protocol published previously (12). Aspirin (100 mg) and clopidogrel (75 mg) or ticlopidine (250 mg) were administered routinely for 3 days before stenting. The procedures were performed for patients with general or local anesthesia. The EPDs (e.g., Angioguard Cordis, RX-ACCUNET Abbott, SpiderRX Ev3, or Filter WireEZ Boston) were used based on the stenting procedure. A suitable balloon was selected based on the ICA diameter, as shown on the DSA image. The use of a balloon (e.g., Aviator, PowerFlex P3, Cordis Endovascular, RX-Viatrac, Abbott, or Invatec) for predilatation or postdilatation depended on the degree of stenosis. Wallstent (159 cases), Precise Cordis (203 cases), Acculink Abbott (136 cases), or Protege Ev3 (72 cases) stents were selected by the neuro-interventionist. The type of stent was selected according to several factors, such as the length and configuration of the lesion, vascular morphology, vessel diameter, plaque characteristics, stent characteristics, and surgeon experience. Postballoon dilatation was only used in patients with unsatisfied stent expansion in the CLS group. All patients were administered aspirin 100 mg/day and clopidogrel 75 mg/day for at least 3 months after the procedure.

Residual stenosis was determined according to CDU detection and DSA imaging within 1 week of and after CAS. Residual stenosis was defined as a stenosis rate ≥30% (13).

### Statistical Analysis

Quantitative data with normal distributions were expressed as the means ± standard deviation (*SD*), such as age, the length/diameter ratio of stents, the diameter and length of balloon for postdilatation, and the length and thickness of plaques. Two-sided *t*-test was used to compare these continuous variables between the residual stenosis group and non-residual stenosis group, and the *t*-value was the statistical value.

The interquartile range [M (P25, P75)] was used to represent the variables that did not conform to a normal distribution, such as the radial and axial expansion rate of stents. The Wilcoxon rank sum test was used to compare these variables between the residual stenosis group and non-residual stenosis group. In addition, the Wilcoxon rank sum test was also used to compare the stent radial expansion rate between CLS and OLS in the residual and in non-residual stenosis groups, separately. In addition, the *Z*-value was the statistical value.

The qualitative data were expressed as percentages or proportions, and comparisons between groups (residual stenosis and non-residual stenosis) were conducted using the Chi-squared test. These variables including male, hypertension, coronary artery disease, diabetes, hyperlipidemia, smoking, family history of stroke, the location of stenosis, degrees of stent length, stent type, plaque morphology (regular shape or irregular shape), calcified, and the incidence of restenosis. In addition, Chi-square test was also used to compare the incidence of residual stenosis, calcified plaques, and irregular plaques between the CLS and OLS groups, and the χ^2^*-*value was the statistical value.

The SPSS software Version 23.0 (IBM, Armonk, NY, USA) was used for statistical analyses. The indicators with *P* < 0.2 in the univariate analysis were entered into a logistic regression analysis to investigate the independent risk factors for residual stenosis. All tests were performed two sided, and a *P* < 0.05 was considered statistically significant. The forest plot was performed using GraphPad Prism 8.0 software (GraphPad Software Inc, San Diego, CA, USA).

## Results

A total of 570 patients (469 men and 101 women) with a mean age of 68 ± 8 years (range, 41–89 years) underwent unilateral CAS. There were 388 (68.1%) patients with hypertension, 194 (34.0%) with diabetes mellitus, 249 (43.7%) with dyslipidemia, 129 (22.6%) with coronary artery disease, 331 (58.1%) smokers, and 18(3.2%) with a family history of stroke.

Among the 570 patients, 130 (22.8%) had residual stenosis, including 122 with residual stenosis < 50% and eight with residual stenosis 50–69%.

A univariate analysis was performed between groups with and without residual stenosis (Table 1). The results revealed that (1) there were no difference in sex, history of coronary heart disease, hyperlipidemia, diabetes mellitus, and location of stenosis between the two groups. (2) There was a higher proportion of irregular and calcified plaques in patients with residual stenosis. Furthermore, patients with surficial calcification plaques had higher residual stenosis rates. The plaque length and thickness were higher in residual stenosis group than non-residual stenosis group (all *P* < 0.05). (3) Of the 570 patients, 159 were treated with CLS and 411 with OLS. Patients with CLS had a higher incidence of residual stenosis than those with OLS (29.5 vs. 20.2%, χ^2^ = 5.71, *P* = 0.01). There was no difference in calcified plaques between the CLS and OLS groups (47.2 vs. 45.9%, χ^2^ = 0.07, *P* = 0.78). There was also no difference in irregular plaques between the CLS and OLS groups (51.6 vs. 45.3%, χ^2^ = 1.83, *P* = 0.17).

**Table 1:** Comparison of factors in groups with and without residual stenosis.

**Variable**	**Residual stenosis**	**Non-residual stenosis**	**Statistical value^a^^,^^b^^,^^c^**	***P-*value**
	**[*n* = 130 (%)]**	**[*n* = 440 (%)]**	**(*t*, χ^**2**^, *Z*)**	
Age (*x* ±*s*, years)	69.65 ± 8.49	67.44 ± 8.28	2.61^a^	0.01
Male	106 (81.5)	363 (82.5)	0.064^b^	0.801
Hypertension	96 (73.8)	292 (66.4)	2.585^b^	0.11
Coronary artery disease	30 (23.1)	99 (22.5)	0.019^b^	0.890
Diabetes	48 (36.9)	146 (33.2)	0.626^b^	0.429
Hyperlipidemia	60 (46.2)	189 (42.9)	0.418^b^	0.518
Smoking	84 (64.6)	247 (56.1)	2.963^b^	0.085
Family history of stroke	7 (5.4)	11 (2.5)	2.73^b^	0.098
Location of stenosis			1.372^b^	0.241
Common carotid artery	7 (33.3)	14 (66.7)		
Internal carotid artery	123 (22.4)	426 (77.6)		
Stent length (cm)			0.646^b^	0.724
L1 (< 4.0 cm)	24 (22.0)	85 (78.0)		
L2 (=4.0 cm)	21 (20.2)	83 (79.8)		
L3 (>4.0 cm)	85 (23.9)	272 (76.1)		
Stent type [*n* (%)]			5.711^b^	0.017
Open cell type	83 (20.2)	328 (79.8)		
Closed cell type	47 (29.5)	112 (70.5)		
Stent (length/diameter) (*x* ±*s*)	7.13 ± 1.81	6.91 ± 1.50	1.277^a^	0.2
Radial expansion rate of stent M (P25, P75)	0.40 (0.35–0.47)	0.61 (0.53–0.69)	14.05^c^	< 0.001
Axial expansion rate of stent	1.07 (1.00–1.23)	1.05 (1.00–1.17)	1.02^c^	0.31
M (P25, P75)				
Postdilatation (*n*, %)	18 (13.8)	55 (12.5)	0.163^b^	0.687
Diameter (*x* ±*s*, mm)	5.17 ± 0.51	5.15 ± 0.62	0.144^a^	0.889
Length (*x* ±*s*, mm)	27.78 ± 5.48	26.55 ± 6.15	0.802^a^	0.428
Restenosis ≥50%	17 (13.1)	9 (2.0)	28.052^b^	< 0.001
Plaque morphology			38.138^b^	< 0.001
Regular shape	38 (12.6)	264 (87.4)		
Irregular shape	92 (34.2)	176 (66.8)		
Calcified plaque			55.099^b^	< 0.001^d^
Non	32 (10.6)	271 (89.4)		
Calcified	98 (36.7)	169 (64.3)	8.505^b^	0.014^e^
Surficial calcification	31 (50.8)	30 (49.2)		
Basal calcification	57 (34.7)	107 (66.3)	4.818^b^	0.028^f^
Internal calcification	10 (23.8)	32 (77.2)		
Plaque length (mm)	22.90 ± 7.11	20.26 ± 5.92	3.856^a^	< 0.001
Plaque thickness (mm)	4.26 ± 1.12	3.91 ± 1.10	3.123^a^	0.002

a *t-value (by two-sided t-test) was the statistical value*.

b *χ^2^ value (by chi-squared test) was the statistical value*.

c *Z-value (by Wilcoxon rank sum test) was the statistical value*.

d *P comparison of the calcified plaque group with the non-calcified plaque group*.

e *P comparison of three types of calcified plaques*.

f *P comparison of the superficial calcification plaque group with the basal calcification plaque group*.

The radial stent expansion rate was lower in the residual stenosis group than in the group without residual stenosis (0.40 vs. 0.61, *P* < 0.001). In addition, we compared the stent radial expansion rate between CLS and OLS in the residual and in non-residual stenosis groups, separately. In the residual stenosis group, the axial expansion rate of CLS was significantly lower than that of the OLS stent (0.380 vs. 0.417, *Z* = 2.03, *P* = 0.04). In the non-residual stenosis group, the axial expansion rate of CLS was lower than that of OLS; however, the difference lacked statistical significance (0.611 vs. 0.618, *Z* = 0.09, *P* = 0.93).

The length-to-diameter ratio inside the stent was higher in the residual stenosis group. There was no difference in stent lengths, postballoon dilatation frequency of performance, balloon size, or axial stent expansion rate between the two groups (all *P* > 0.05).

**Figure 2:**
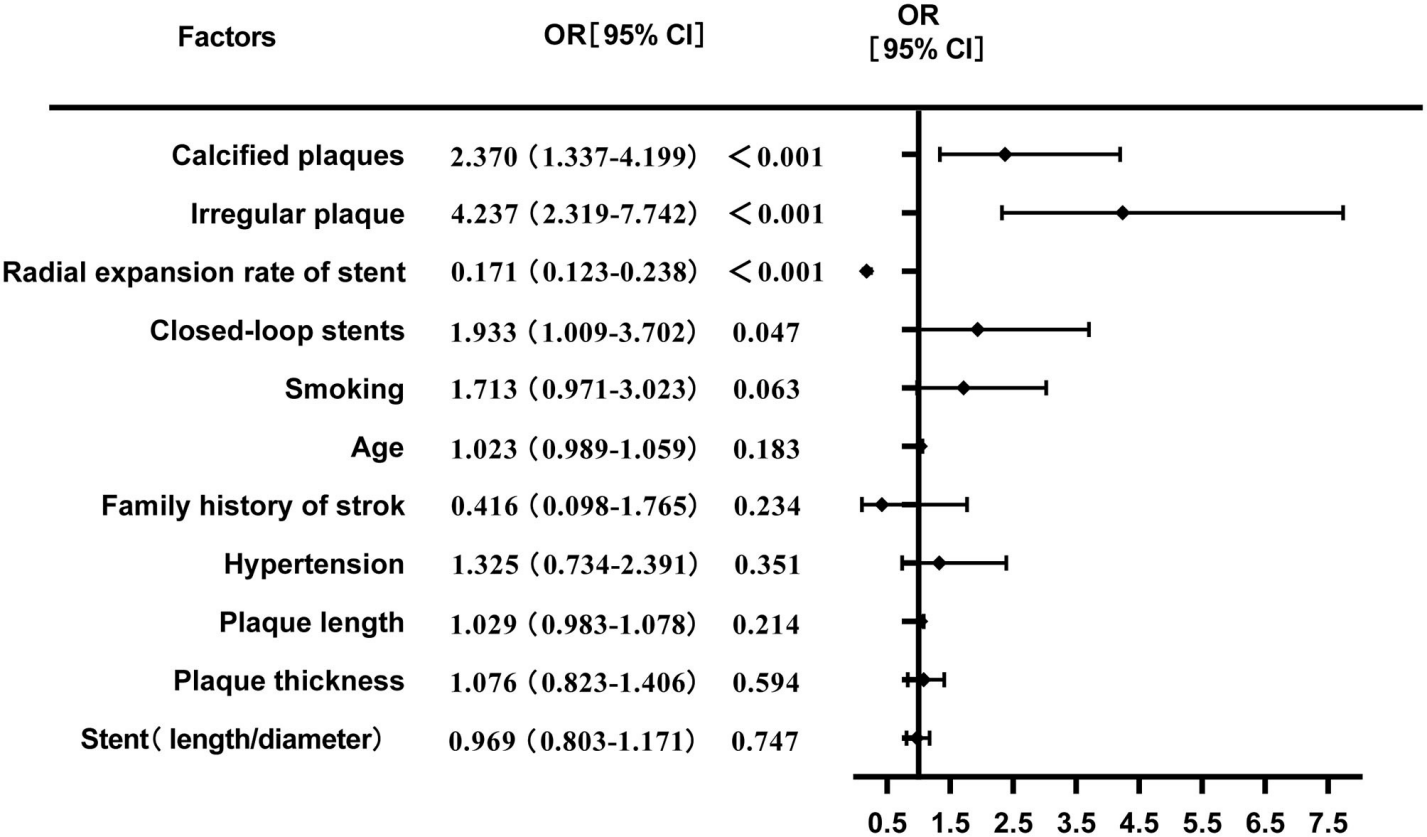
Multivariate logistic regression analysis of risk factors for residual stenosis after CAS.

The multivariate logistic regression analysis showed that irregular plaques, calcified plaques, stent type (closed-loop stent), and lower radial expansion rate were independent risk factors for residual stenosis after CAS (*P* < 0.05). Although the *P*-values of age, hypertension, smoking, family history of stroke, the length-to-diameter ratio, and the length and thickness of plaques were < 0.2 in the univariate analysis, the logistic regression analysis showed that they were not independent risk factors for residual stenosis (*P* > 0.05) (Table 2; Figure 2).

**Table 2:** Logistic regression analysis of factors for residual stenosis after CAS.

**Factors**	**OR**	**95%CI**	***P*-value**
Calcified plaques	2.370	1.337–4.199	< 0.001
Irregular plaques	4.237	2.319–7.742	< 0.001
Radial expansion rate of stent	0.171	0.123–0.238	< 0.001
Closed cell stents	1.933	1.009–3.702	0.047
Smoking	1.713	0.971–3.023	0.063
Age	1.023	0.989–1.059	0.183
Family history of stroke	0.416	0.098–1.765	0.234
Hypertension	1.325	0.734–2.391	0.351
Plaque length	1.029	0.983–1.078	0.214
Plaque thickness	1.076	0.823–1.406	0.594
Stent (length/diameter)	0.969	0.803–1.171	0.747

Finally, among the 570 patients, 476 were followed up for a mean duration of 2.1 ± 1.7 years (range, 1–6 years), and the lost to follow-up rate was 16.5%. Restenosis occurred in 25 patients (5.25%), and the incidence of restenosis in the residual stenosis group was higher than that in the group without residual stenosis (13.1 vs. 2.0%, χ^2^ = 28.05, *P* < 0.001). A second operation was performed in two patients [one with CEA and one with percutaneous transluminal angioplasty (PTA)].

## Discussion

Recent clinical studies have gradually confirmed that there is no significant difference in the perioperative stroke, myocardial infarction, and fatality rates between CAS and CEA cohorts. Furthermore, the outcomes in the postprocedural period after CAS and CEA were similar over long-term follow-ups (14). Recent studies have shown that CAS has become an effective method for treating patients with severe carotid atherosclerotic stenosis (15). A major issue with CAS is the occurrence of in-stent neointimal proliferation, which may result in restenosis of the stented vessel. However, a previous study showed that restenosis is likely to occur after more than 12 months, due to the thickness of the in-stent neointima (16). Focused evaluations of residual stenosis with regular follow-ups will reduce restenosis rates.

Our previous studies confirmed that the stent type was related to restenosis after CAS and that implantation of closed-cell stents was an independent risk factor for restenosis after CAS (17). Its strong radial strength, poor flexibility, and poor adherence to the vascular wall may cause residual stenosis after stent implantation. The present study showed that the radial expansion rate of closed-cell stents was significantly lower than that of open-cell stents.

Furthermore, open-cell and closed-cell stents have different mechanical properties, which might influence the risk of residual or recurrent stenosis after treatment. One potential advantage of open-cell stents is that they are more flexible and better suited for tortuous vessels. On the contrary, closed-cell stents have tighter meshes, which may provide better coverage of the atheromatous lesion but are consequently more rigid. A recent study found that, compared with open-cell stents, the more rigid and more densely packed material in closed-cell stents might lead to greater irritation of the vessel wall, which in turn might stimulate neointima hyperplasia and result in a higher occurrence of restenosis (18). In this study, we found that the incidence of residual stenosis was higher for closed-cell stents than for open-cell stents, and different stent lengths did not influence residual stenosis. A few previous studies found that the closed-cell stents used for unstable plaques may not solve tissue prolapse, and postdilatation during CAS for unstable or calcified plaques decreases in-stent large tissue prolapse and reduces the restenosis rates (19, 20). However, this study did not analyze the impact of stent materials and intraoperative predilatation procedures on residual stenosis, which will be further investigated in subsequent studies.

In addition, previous studies have reported that the nature of plaques is an important factor that influences stent insufficiency. As early as 1999, studies have shown that severe plaque calcification is a risk factor for coronary artery stent insufficiency (21). The present study found that morphological irregular plaques and calcified plaques were independent risk factors for residual stenosis after CAS. In this study, calcified plaques were further categorized as superficial, basal, and internal calcifications. The residual stenosis rate was highest in the basal calcification group. Irregular plaques can lead to poor stent and lumen adhesion, incomplete stent expansion, and residual stenosis (22). Previous studies also found that it is still difficult to restore fibrous cap integrity after stent implantation; therefore, both the occurrence of long-term restenosis and recent cerebral infarction after stent implantation are related to these properties. Using a closed-cell stent-in-stent technique may result in smaller free-cell area than open-cell stents, which restricts plaque protrusion and, for unstable plaques, may prevent plaque protrusion and ischemic complications (23–25).

Residual stenosis after CAS is closely related to the occurrence of long-term restenosis and has a significant influence on long-term patient prognosis (26, 27). Evaluating the structural characteristics of severe carotid artery stenosis before CAS by CDU helps clinicians select the appropriate stent type. In addition, regular follow-up of the changes in hemodynamics and blood vessel structures after CAS revascularization and stent implantation can identify residual stenosis early, and early treatment will reduce the incidence of restenosis, which is particularly important for improving long-term efficacy. In this study, the correlation between the angulation of the stent and the lumen, intraoperative balloon dilation pressure, and residual stenosis after CAS were not evaluated, and these factors need further study.

## Conclusions

In conclusion, irregular and calcified plaques are risk factors for residual stenosis after CAS. In addition, a closed cell stent and poor radial expansion increase the risk of residual stenosis.

## Data Availability Statement

The raw data supporting the conclusions of this article will be made available by the authors, without undue reservation.

## Author Contributions

YT and YH: manuscript editing, drafting and reviewing, data analysis, and study design. LJia and BL: case materials and study reviewers. LJiao: surgery of carotid artery stent and the operation guide. All authors contributed to the article and approved the submitted version.

## Conflict of Interest

The authors declare that the research was conducted in the absence of any commercial or financial relationships that could be construed as a potential conflict of interest.

## References

[1] Perkins WJ Lanzino G Brott TG. Carotid stenting vs. endarterectomy: new results in perspective. Mayo Clin Proc. (2010) 85:1101–8. 10.4065/mcp.2010.0588 21123637 PMC2996157

[2] Yip HK Sunq PH Wu CJ Yu CM. Carotid stenting and endarterectomy. Int J Cardiol. (2016) 214:166–74. 10.1016/j.ijcard.2016.03.172 27061654

[3] Brott TG Hobson RW Howard G Roubin GS Clark WM Brooks W. Stenting vs. endarterectomy for treatment of carotid-artery stenosis. N Engl J Med. (2010) 363:11–23. 10.1056/NEJMoa0912321 20505173 PMC2932446

[4] Brott TG Calvet D Howard G Gregson J Algra A Becquemin J. Long-term outcomes of stenting and endarterectomy for symptomatic carotid stenosis: a preplanned pooled analysis of individual patient data. Lancet Neurol. (2019) 18:348–56. 10.1016/S1474-4422(19)30028-6 30738706 PMC6773606

[5] Lal BK Beach KW Roubin GS Lutsep HL Moore WS Malas MB. Restenosis after carotid artery stenting and endarterectomy: a secondary analysis of CREST, a randomised controlled trial. Lancer Neurol. (2012) 11:755–63. 10.1016/S1474-4422(12)70159-X 22857850 PMC3912998

[6] Xin WQ Li MQ Li K Li QF Zhao Y Wang WH. Systematic and comprehensive comparison of incidence of restenosis between carotid endarterectomy and carotid artery stenting in the patients with atherosclerotic carotid stenosis. World Neurosurg. (2019) 125:74–86. 10.1016/j.wneu.2019.01.118 30710719

[7] Cosottini M Michelassi MC Bencivelli W Lazzarotti G Picchietti S Orlandi G. In stent restenosis predictors after carotid artery stenting. Stroke Res Treat. (2010) 2010:864724. 10.4061/2010/864724 20798894 PMC2925310

[8] Doau B Chalouhi N Starke RM Dalyai R Polifka A Sarkar K. Predictors of restenosis after carotid artery stenting in 241 cases. J Neurointerv Surg. (2015) 8:677–9. 10.1136/neurintsurg-2015-011783 26338829

[9] Chinese Medical Doctor Association of Ultrasonography Guideline of Vascular Ultrasound Examination. Beijing: People's Military Medical Press (2011).

[10] Zhou C Liu CH Qiao T. Carotid artery calcification score and its association with cognitive impairment. Clin Interv Aging. (2019) 14:167–77. 10.2147/CIA.S192586 30697041 PMC6342141

[11] Bladin CF Alexandrov AV Murphy J Maggisano R Norris JW. Carotid Stenosis Index. A new method of measuring internal carotid artery stenosis. Stroke. (1995) 26:230–4. 10.1161/01.str.26.2.230 7831693

[12] Jiao LQ Song G Li SM Miao ZR Zhu FS Ji XM. Thirty-day outcome of carotid artery stenting in Chinese patients: a single-center experience. Chin Med J. (2013) 126:3915–20. 10.3760/cma.j.issn.0366-6999.20131870 24157156

[13] Bates ER Babb JD Casey DE Cates CU Duckwiler GR Feldman TE. For American College of Cardiology Foundation, American Society of Interventional and Therapeutic Neurotadiology, Society for Vascular Medicine and Biology, Society of Interventional Radiology. ACCF/SCAI/SVMB/SIR/ASITN 2007 clinical expert consensus document on carotid stenting: a report of the American College of Cardiology Foundation Task Force on Clinical Expert Consensus Documents (ACCF/SCAI/SVMB/SIR/ASITN Clinical Expert Consensus Document Committee on Carotid Stenting. J Am Coll Cardiol. (2007) 49:126–70. 10.1016/j.jacc.2006.10.021 17207736

[14] Bonati LH Dobson J Featherstone RL Ederle J van de Worp HB de Borst GJ. Long-term outcomes after stenting vs. endarterectomy for treatment of symptomatic carotid stenosis: the International Carotid Stenting Study (ICSS) randomized trial. Lancet. (2015) 385:529–38. 10.1016/S0140-6736(14)61184-3 25453443 PMC4322188

[15] Brott TG Howard G Roubin GS Meschia JF Mackey A Brooks W. Long-term results of stenting vs. endarterectomy for carotid artery stenosis. N Engl J Med. (2016) 374:1021–31. 10.1056/NEJMoa1505215 26890472 PMC4874663

[16] Yamashita K Kokuzawa J Kuroad T Murase S Kumagai M Kaku Y. In-stent hypodense area at 2 weeks following carotid artery stenting predicts neointimal hyperplasia after 2 years. Neurotadiol J. (2018) 31:280–7. 10.1177/1971400917727006 28816615 PMC5958497

[17] Tang Y Hua Y Jia LY Wang LL Zhao XY Zhou YH Restenosis after carotid artery stenting was followed up with ultrasound and the analysis of its influencing factors. Chin J Cerebrovasc Dis. (2012) 9:564–8. 10.3969/j.issn.1672-5921.2012.11.002

[18] Muller MD Gregson J McCabe DJH Nederkoorn PJ van der Worp HB Borst GJ. Stent design, restenosis and recurrent stroke after carotid artery stenting in the international carotid stenting study. Stroke. (2019) 50:3013–20. 10.1161/STROKEAHA.118.024076 31547798

[19] Harada K Oshikata S Kajihara M. Optical coherence tomography evaluation of tissue prolapse after carotid artery stenting using closed cell design stents for unstable plaque. J Neurointery Surg. (2018) 10:229–34. 10.1136/neurintsurg-2017-013004 28360353

[20] Harada K Kajihara M Sankoda Y Taniguchi S. Efficacy of post-dilatation during carotid artery stenting for unstable plaque using closed-cell design stents evaluated by optical coherence tomography. J Neurotadiol. (2019) 46:384–9. 10.1016/j.neurad.2019.03.006 30954551

[21] Henneke KH Regar E Konig A Werner F Klauss V Metz J. Impact of targer-lesion calcification on coronary stent expansion after rotational atherectomy. Am Heart J. (1999) 137:93–9. 10.1016/s0002-8703(99)70463-1 9878940

[22] Katano H Mase M Nishikawa Y Yamada K. Surgical treatment for carotid stenosis with highly calcified palques. J Stroke Cerebrovasc Dis. (2014) 23:148–54. 10.1016/j.jstrokecerebrovas2012.11.019 23273787

[23] Kohyama S Kazekaka K Iko M Aikawa H Tsutsumi M Go Y. Spontaneous improvement of persistent ulceration after carotid artery stenting. AJNR Am J Neuroadiol. (2006) 27:151–6. 16418376 PMC7976077

[24] Misaki K Uchiyama N Mohri M Hayashi Y Ueda F Nakada M. Prediction of carotid artery in-stent restenosis by quantitative assessment of vulnerable plaque using computed tomography. J Neurotadiol. (2016) 43:18–24. 10.1016/j.neurad.2015.09.002 26603106

[25] Myouchin K Takayama K Wada T Miyasaka T Tanaka T Kotsugi M. Carotid artery stenting using a closed-cell stent-in-stent technique for unstable plaque. J Endovasc Ther. (2019) 26:565–71. 10.1177/1526602819847698 31074315

[26] Bonati LH Ederle J Dobson J Engelter S Featherstone RL Gaines PA The length of carotid stenosis predicts peri-procedural stroke or death and restenosis in patients randomized to endovascular treatment or endarterectomy. Int J Stroke. (2014) 9:297–305. 10.1111/ijs.12084 23895672 PMC4232022

[27] Liu Y Hua Y Liu R Wang L Duan C Ling C. Ultrasonographical features associated with the progression of atherosclerosis in patients with moderate internal carotid artery stenosis. Transl Stroke Res. (2018) 9:375–81. 10.1007/s12975-017-0592-9 29196884

